# Species-specific Posture of Human Foetus in Late First Trimester

**DOI:** 10.1038/s41598-017-18384-w

**Published:** 2018-01-08

**Authors:** Yoshiyuki Ohmura, Seiichi Morokuma, Kiyoko Kato, Yasuo Kuniyoshi

**Affiliations:** 1 Department of Mechano-Informatics, Graduate School of Information Science and Technology, The University of Tokyo 7-3-1, Hongo, Bunkyo-ku, Tokyo, Japan; 2 Research Centre for Environmental and Developmental Medical Sciences, Kyushu University, Fukuoka, Japan; 3 Department of Obstetrics and Gynecology, Kyushu University hospital, Fukuoka, Japan

## Abstract

The ontogeny associated with the arm-hanging posture, which is considered ape-specific, remains unknown. To examine its ontogeny, we measured foetal movements of 62 human foetuses aged 10–20 gestation weeks using four-dimensional sonography. We observed that the first-trimester foetuses show this particular species-specific posture. After 11 weeks of gestation, all foetuses showed the arm-hanging posture, and the posture was most frequently observed at 14–16 weeks of gestation. Moreover, this posture often involved extension of both arms and both legs, indicating that it is not myogenic but neurogenic. Furthermore, early ontogeny suggests that it originates because of subcortical activity. Such posture extension bias and persistence indicates that vestibulospinal tract maturation involves the ontogeny of arm-hanging posture during 14–16 weeks of gestation.

## Introduction

The arm-hanging posture is the most distinctive feature among apes^1^; however, its ontogeny remains unknown. In humans, studies to understand the functional maturation of the central nervous system are limited to non-invasive measurement. Therefore, the ontogeny of several behaviours during foetal period provides important information for understanding neural function because the development of nervous system is correlated with the appearance of behaviours; moreover, the maturational stages vary for different brain regions.

Human foetuses spend a significant amount of time in the flexion posture^2,3^. The flexion of the distal forearm frequently occurs after 12 weeks of gestation^2^, regardless of sufficient space for movement^4^, and the flexion predominance lasts until few months after birth^5^. The flexion predominance during the foetal period indicates that the ape-specific arm-hanging posture, which requires extension of both shoulders and both elbows, develops postnatally; however, several species-specific movements can be observed during the foetal period in rodents^6^ and humans^4,7,8^. The temporary disappearance of a pattern and its re-emergence is a common phenomenon during behavioural development^6,9^. Therefore, we hypothesised that the ape-specific arm-hanging posture prenatally develops before the development of flexion predominance. To examine this issue, we measured the foetal movements of 62 foetuses aged 10–20 gestation weeks using four-dimensional (4D) sonography^10,11^. Subsequently, we observed that all foetuses showed arm-hanging-like posture after 11 weeks of gestation. To the best of our knowledge, this is the first study that reports that first-trimester foetuses can demonstrate species-specific posture.

## Results

### Observations

**Figure 1:**
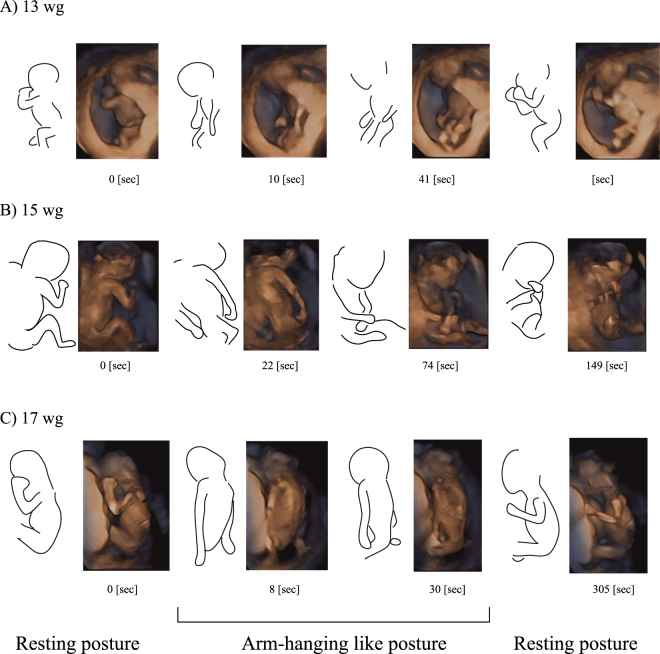
Example of arm-hanging-like posture sequences. Outline of foetuses and sonography images. wg = weeks of gestation.

**Figure 2:**
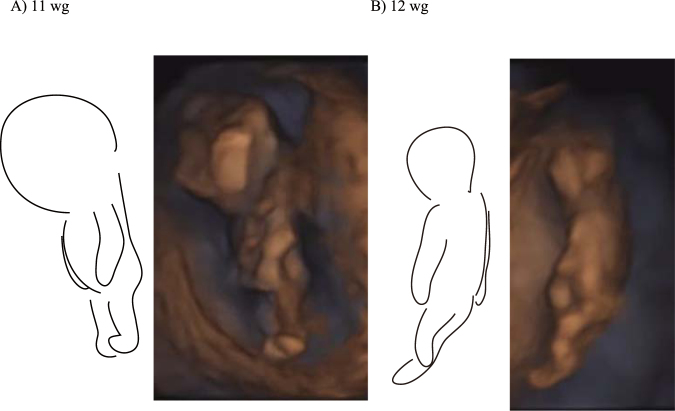
Example of standing-like posture.

We observed that all foetuses after 11 weeks of gestation showed arm-hanging-like posture; this posture was characterised by the extension of bilateral shoulders and elbows. Once foetuses showed the arm-hanging-like posture, they maintained it for an average of 38.26 ± 37.96 s. Figure 1 shows few examples of the arm-hanging-like posture sequence (see also Supplementary Video). We frequently observed that these postures were initialised by a synchronous bilateral extension of both arms and ended after reaching the resting flexion posture. During the arm-hanging-like posture, the trunk or leg extension behaviours were frequently observed; however, arm movement was relatively less frequent. Moreover, we occasionally observed a standing-like posture (Fig. 2); however, this posture was not observed after 14 weeks of gestation because of the spatial restriction within the womb. Upon extension of the legs, the neck, hip and trunk of the foetuses were frequently bent because of their contact with the womb walls.

**Figure 3:**
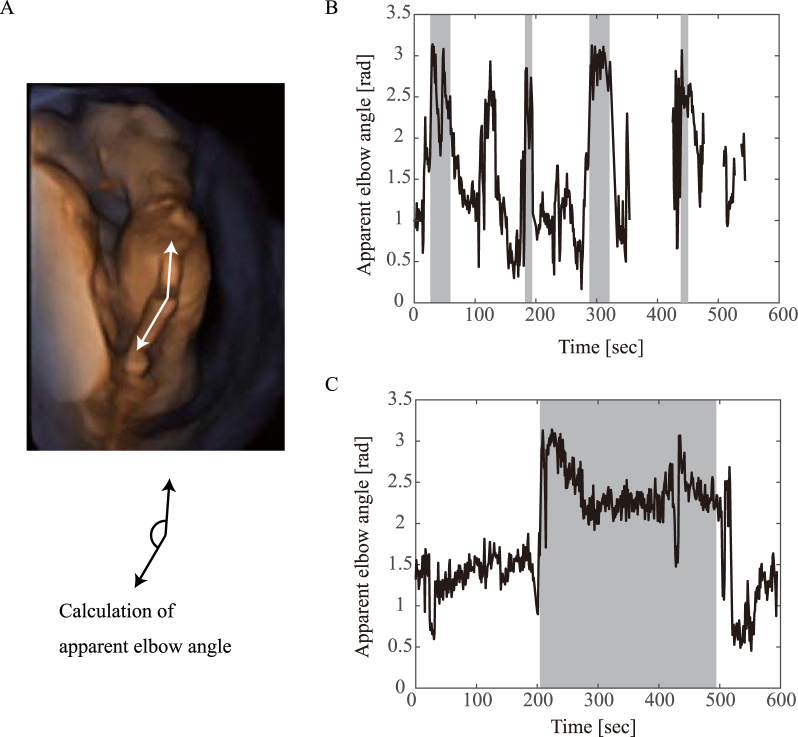
Examples of elbow angle appearance trajectories. (**A**) The elbow angles were calculated from two lines: a line from the elbow position to the wrist position and another line from the elbow position to the shoulder position in each video frame. (**B**) Example from a foetus at 14 weeks of gestation. (**C**) Example from a foetus at 17 weeks of gestation. Grey shading indicates the period of arm-hanging-like posture.

To validate our observations, we calculated the elbow angle appearance trajectories (Fig. 3 and Supplementary Figure S1). Because the elbow angle appearance is affected by foetal posture as well as camera angles, we selected trackable videos considering the camera angle stability and minimal changes in the foetal posture. We then calculated the elbow angles using two lines: a line from the elbow position to the shoulder position, and a line from the elbow position to the wrist position (Fig. 3a). As shown in Fig. 3, the elbow angle trajectories showed slower frequency dynamics than that of the gross movements.

### Quantitative analysis of frequency

**Figure 4:**
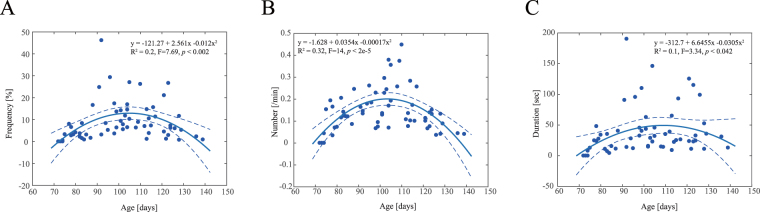
Scatter plot and polynomial regression analysis of the arm-hanging-like posture frequency. (**A**) Age-related changes in arm-hanging-like posture frequency. (**B**) Age-related changes in occurrences per minute of arm-hanging-like posture. (**C**) Age-related changes in the arm-hanging-like posture duration. The error bar indicates 95% confidence interval.

We quantified the frequency of the arm-hanging-like posture in 62 foetuses aged 10–20 gestation weeks. We discriminated each moment to identify the arm-hanging-like posture. In cases where we were unable to clearly determine the classification because of unclear visualisation on sonography or bad foetal postures, we removed specific durations from the analysis. Foetuses aged 10 weeks rarely showed the arm-hanging posture. However, all foetuses after 11 weeks of gestation showed the posture. The average frequency of the posture was 8.9% ± 8.8%. Moreover, we also observed age-dependent changes in frequency (F = 7.69, *P* < 0.002, Fig. 4a). We confirmed that intra-observer reliability did not account for age-dependent changes (Supplementary Figure S2). Regression analysis revealed that the frequency of the arm-hanging-like posture changed with age, indicated by an inverted U-shaped curve. The occurrences per minute (F = 14, *P* < 2^e-5^) and the duration (F = 3.34, *P* < 0.042) also changed with age, indicated by an inverted U-shaped curve (Fig. 4b and c). A peak was attained during 104–110 days (14–16 weeks of gestation).

## Discussion

In the present study, we observed that human foetuses frequently showed an ape-specific arm-hanging-like posture after 11 weeks of gestation. This posture persisted for 40 s on average. The frequency of the posture peaked during 14–16 weeks of gestation.

Based on literature review, this is the first study that demonstrates that the late first-trimester foetuses can consistently show ape-specific postures. Until now, the arm-hanging posture had not been recognised as a discriminable spontaneous movement; however, the posture can be clearly observed through the following features: first, the gross arm movements are rarely seen during the arm-hanging posture; second, the arm-hanging posture is frequently initiated by bilateral simultaneous extension of shoulders and elbows; finally, the arm-hanging posture is slowly ended after reaching the resting flexion posture. These stereotypes sequences can be used to discriminate the arm-hanging posture. Moreover, the arm-hanging posture observed in this study differs from the spontaneous movements observed in other previous studies^4,7,8,10–29^. Hence, general movements^4,7,8,22–25,27–29^ involve the entire body with a variable sequence of arm, leg, neck and trunk movements; however, in our study, both arms persistently extended without any gross movements during the arm-hanging posture. This observation supports the idea that the arm-hanging posture differs from general movements. Moreover, the arm-hanging posture also differs from the isolated limb movements^4,7,8,10–29^ because the entire body extension bias exists during the arm-hanging posture.

During the arm-hanging posture, the foetus frequently extended both legs, indicating that all extensor body muscles are simultaneously activated during this period. Such synchronous activation indicates that this activity is not myogenic but neurogenic. Furthermore, early ontogeny suggests that the arm-hanging posture is organised by the subcortical system because the corticospinal tract is immature during the early foetal period^30^. We speculate that the arm-hanging posture development is related to the vestibulospinal tract development, which involves axial and proximal slow-twitch muscle extension^31,32^. Although the vestibulospinal tract development remains mostly unknown in humans, anatomical evidence^33–35^ indicates that the vestibulospinal tract is relatively matured during the early foetal period.

Visualising whole body movements by 4D sonography after 20 weeks of gestation is difficult because of limited field angles^11^. Therefore, we were unable to examine the arm-hanging posture frequency after this period. According to previous studies^2,3^, elbow extension is rarely seen after 20 weeks of gestation. Collectively, the data suggest that the arm-hanging posture frequency peaks at 14–16 weeks of gestation and decreases thereafter. This early peak occurrence and decrease suggests that the age-dependent arm-hanging posture decrease must be interpreted as an expression of neuromaturation rather than expression of environmental restriction because the space within the womb is sufficient to enable movement at this period^2–4^. We speculated that the vestibulospinal tract maturation will contribute to the arm extension posture decrease because spontaneous patterned activity is considered a general transient feature of developing neural circuits in several brain regions^36^.

In conclusion, the present study suggests that the ape-specific arm-hanging posture develops at the late first trimester, but not during the postnatal period, indicating that the ape-specific behaviour is organised by the subcortical nervous system.

## Methods

### Foetal population

The study population consisted of 62 normal singleton pregnant women at 10–20 weeks of gestation that received perinatal management at the Maternity and Prenatal Care Unit, Kyushu University Hospital. This cross-sectional study was conducted between April 2013 and January 2017. None of the selected cases featured foetal abnormalities (i.e. morphological defects and foetal hypoplasia) or maternal diseases. The clinical characteristics of the recruited subjects are indicated in Table 1. No obvious maternal complications were observed. The gestational age was calculated from the mother’s last menstrual cycle and confirmed during the first trimester via serial ultrasonographic measurements of the crown–rump length.

**Table 1:** Clinical characteristics of the recruited participants.

Maternal age	35 (21–43)
Gestational age at delivery (weeks.days)	38.5 (36–41)
Birth-weight (g)	3010 (2215–3885)
Apgar score	
1 min	9 (8–9)
5 min	9 (9–10)
pH of the umbilical artery	7.30 (7.10–7.41)

Median data are depicted in ranges.

The present study was designed according to the Declaration of Helsinki’s ethical principles and was approved by the Kyushu University institutional review board (No. 27–51) and the Life Science Research Ethics and Safety, the University of Tokyo ethical committee (17–21). Informed consent was obtained from all mothers participating in the study, prior to initiation.

### Procedure

The participants were placed in a semi-recumbent position in a quiet room with a relatively low illumination. The study was conducted between 13:00 and 16:00, at least 2 h after food ingestion.

The entire foetus was monitored using transabdominal 4D ultrasonography (Voluson E8 Expert; GE Healthcare, Kretz, Zipf, Austria) with a curved array transabdominal transducer (4–8.5 MHz). After a standard 2D sonographic assessment, the device was switched to the 4D mode, and the transducer was positioned over the foetus to observe foetal movement. The foetal movement observation was performed for more than 30 min at a frame rate of ≥4 frames/s. Data were recorded in an MP4 digital video file format (30 frames/s) on a memory card.

### Elbow angle appearance

We calculated elbow angles at least once every 15 video frames (i.e. 2 Hz) to visualise the foetal elbow movement. The elbow angle was calculated using two lines: a line from the elbow position to the shoulder position and another line from the elbow position to the wrist position. These positions were manually tracked in the digital video. The software for tracking these joint positions was developed by one of the authors. When one of the joint positions was unclear, the elbow angle was not calculated. Because the elbow angle appearance was affected by the foetal posture and camera angle, the calculation of elbow appearance trajectories was conducted using only segmented video files, which showed a relatively stable camera angle and small change in foetal posture.

### Analysis of frequency of arm-hanging posture

To examine the developmental change of the arm-hanging posture frequency, we calculated the ratio of time length spent in this posture to the total measurement duration. We classified each video file frame into three categories, namely, arm-hanging state, not arm-hanging state, or undetermined state, by the subtitle maker function of the authoring software, TMPGEnc Authoring Works 5. When we determined the bilateral extension of both shoulders and elbows from the hand position, which were always around the waist or hip by extension of both shoulders and elbows, we classified the frame into an arm-hanging state. When one arm was invisible because of occlusion, we estimated the hand position from this context. We then defined the ratio of the frame number of arm-hanging state to the frame number of both arm-hanging and not arm-hanging states as the frequency of the arm-hanging posture. This procedure was conducted for all foetal videos. We also calculated the occurrences per minute and the mean duration of the arm-hanging state. We also examined inter- and intra-observer reliabilities (Supplementary Information).

### Statistical analysis

Stepwise polynomial regression analysis, with foetal age as the dependent variable and frequency, occurrences per minute and duration of arm-hanging-like posture as independent variables, was used to describe the best-fit formula. Polynomial expressions were considered up to the fourth degree. We calculated the F-statistics for comparison between the final regression and constant model. *P* < 0.05 was considered statistically significant. The statistical tests were performed using the MATLAB R2016b Statistics Toolbox (MathWorks Japan, Tokyo, Japan).

### Data availability

All data generated during and/or analysed during the current study are available from the corresponding author upon reasonable request.

## Electronic supplementary material

Example of arm-hanging-like posture

Supplementary Information
