# Supplementary material for: Species-specific Posture of Human Foetus in Late First Trimester
Source: Sci Rep. 2018 Jan 8;8:27. doi: 10.1038/s41598-017-18384-w (PMC5758525; doi:10.1038/s41598-017-18384-w)
Supplement: Supplementary file 2 — Supplementary Information [file 41598_2017_18384_MOESM2_ESM.pdf]

Species-specific posture of human foetus in late first trimester

***Yoshiyuki Ohmura[1], Seiichi Morokuma[2, 3], Kiyoko Kato[3], Yasuo Kuniyoshi[1]**

[1] Department of Mechano-Informatics, Graduate School of Information Science and Technology, The University of Tokyo 7-3-1, Hongo, Bunkyo-ku, Tokyo, Japan

[2] Research Center for Environmental and Developmental Medical Sciences, Kyushu University, Fukuoka, Japan

[3]Department of Obstetrics and Gynecology, Kyushu University hospital, Fukuoka, Japan

* Corresponding author: E-mail: ohmura@isi.imi.i.u-tokyo.ac.jp

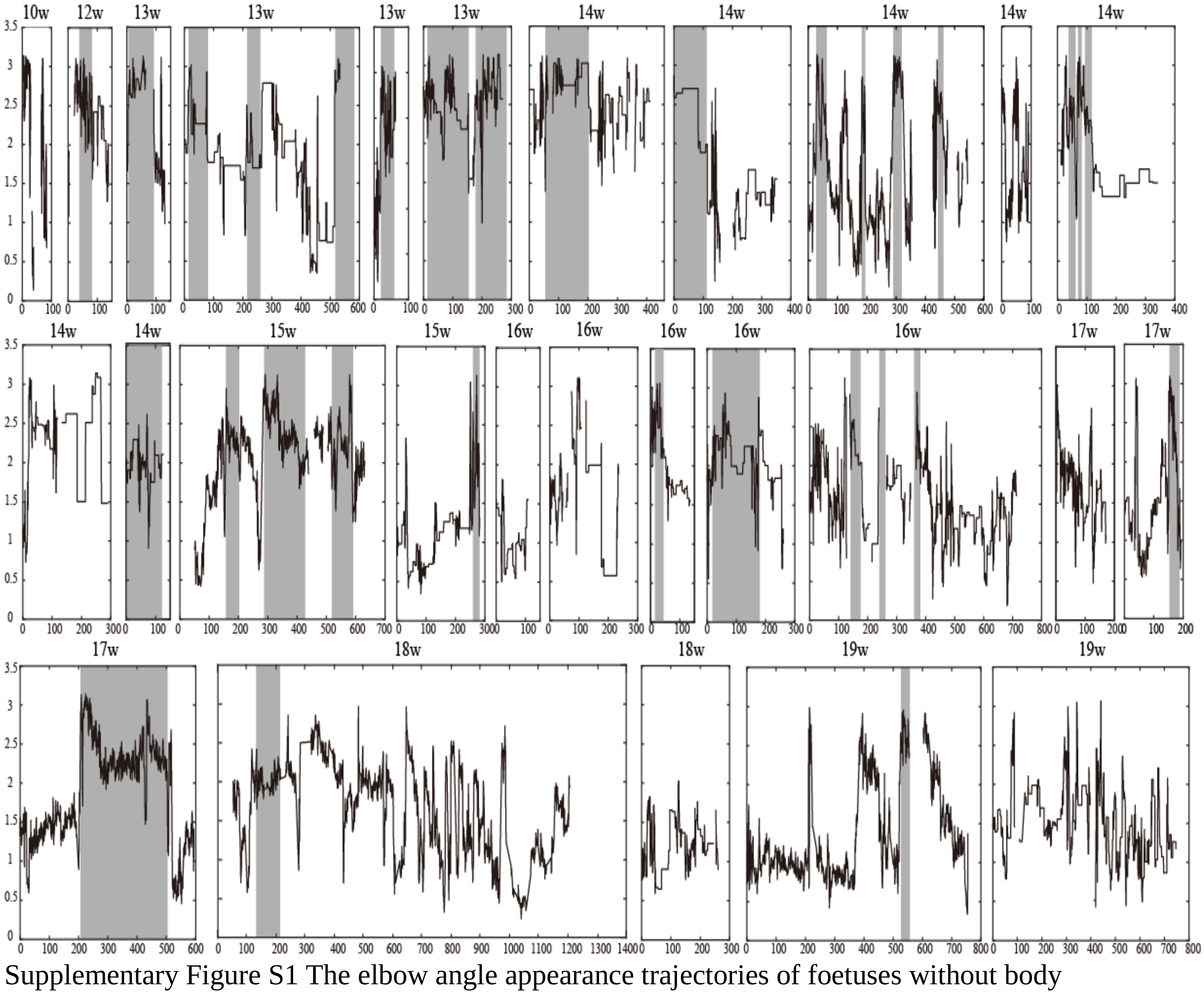

Supplementary Figure S1 The elbow angle appearance trajectories of foetuses without body rolling motion. Gray shading indicates the period of arm-hanging-like posture.

Examination of intra-observer error

To eliminate the possibility of age-related change in frequency of arm-hanging posture being affected by intra-observer reliability, we examined the intra-observer error. One observer (Y.O.) repeated the classification of each frame of video by same method. We randomly selected 32 participants. Time interval between repeated measurements was six months. Consequently, the intra-observer error in the frequency of arm-hanging posture was not correlated with age ($F = 0.968$, $p > 0.3$, quadric, $n = 32$), indicating that intra-observer reliability did not affect the result of age-related change.

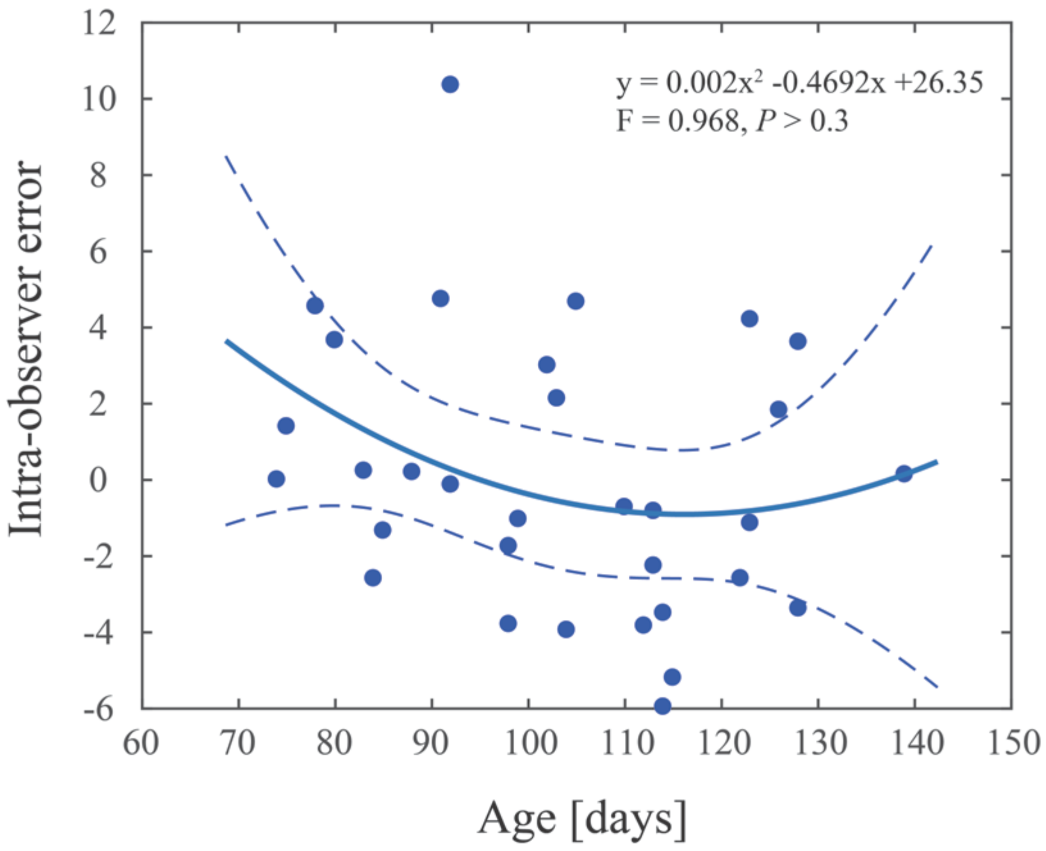

Supplementary Figure S2 Examination of intra-observer error

Examination of inter-observer error

Two observers (Y.O. and S.M.) classified each frame of video by same method. We randomly selected 6 participants. Consequently, Cohen's kappa [S1] was 0.78.

Supplementary Table S1 Frequency data analysed in this study

Foetal age (days)	Measurement time (sec)	Time length in undetermined state (sec)	Time length in arm-hanging state (sec)	Number of occurrences of arm-hanging state
128	2501	694	210	4
136	1904	546	31	1
83	2165	475	141	4
77	2204	655	124	5
113	2526	2084	65	1
82	2915	844	44	4
74	2498	578	0	0
72	1785	1029	0	0
114	2312	550	67	3
74	1876	507	8	1
123	2295	569	460	4
116	2421	633	279	3
139	2154	683	13	1
117	2110	475	185	7
78	2046	230	119	5
79	1913	200	55	2
75	2462	505	64	5
78	1984	235	48	1
92	2572	923	761	4
82	2531	192	86	8
113	2712	1144	18	2
73	2118	599	0	0
122	2271	459	22	2
121	2914	1129	376	3
80	1565	745	34	1
101	1591	659	87	2
91	1549	451	272	3
99	2119	506	220	2
114	1670	822	89	4
105	2053	872	319	7
102	2141	924	95	6
96	2690	734	573	6
112	2044	371	117	3
104	2953	1227	292	2
85	2199	210	62	4
121	1812	228	71	3

123	2023	442	46	2
124	2318	728	98	4
115	2070	334	126	5
109	2240	796	128	9
88	2308	356	324	8
104	2041	274	114	5
110	2169	429	456	13
108	2326	1393	59	4
98	2803	1005	65	2
87	2675	580	34	3
98	2124	290	318	7
104	2444	544	276	12
101	2033	265	251	4
99	2117	213	121	3
83	2198	260	29	4
92	2243	452	56	4
102	3018	1177	102	7
103	2365	648	202	7
95	3089	1253	96	8
84	2204	238	17	4
94	2308	631	140	5
105	2348	790	160	5
129	2718	684	107	3
126	2467	816	99	1
124	2304	483	131	4
128	1865	406	11	1

Reference

[S1] Cohen, J. A coefficient of agreement for nominal scales. *Educ. and Psychol. Meat.* **20**, 37-46 (1960).
